# Induction by Phenobarbital of Phase I and II Xenobiotic-Metabolizing Enzymes in Bovine Liver: An Overall Catalytic and Immunochemical Characterization

**DOI:** 10.3390/ijms23073564

**Published:** 2022-03-24

**Authors:** Michela Cantiello, Monica Carletti, Mery Giantin, Giulia Gardini, Francesca Capolongo, Paolo Cascio, Marianna Pauletto, Flavia Girolami, Mauro Dacasto, Carlo Nebbia

**Affiliations:** 1 Department of Veterinary Sciences, University of Turin, 10095 Grugliasco, Italy; michela.cantiello@gmail.com (M.C.); monica.carletti@irta-ricerche.it (M.C.); giulia.gardini@unito.it (G.G.); paolo.cascio@unito.it (P.C.); flavia.girolami@unito.it (F.G.); 2 Department of Comparative Biomedicine and Food Science, University of Padua, 35020 Agripolis Legnaro, Italy; mery.giantin@unipd.it (M.G.); francesca.capolongo@unipd.it (F.C.); marianna.pauletto@unipd.it (M.P.)

**Keywords:** cattle, drug-metabolizing enzymes, hepatic drug metabolism, enzyme activity, induction, phenobarbital, species differences

## Abstract

In cattle, phenobarbital (PB) upregulates target drug-metabolizing enzyme (DME) mRNA levels. However, few data about PB’s post-transcriptional effects are actually available. This work provides the first, and an almost complete, characterization of PB-dependent changes in DME catalytic activities in bovine liver using common probe substrates and confirmatory immunoblotting investigations. As expected, PB increased the total cytochrome P450 (CYP) content and the extent of metyrapone binding; moreover, an augmentation of protein amounts and related enzyme activities was observed for known PB targets such as CYP2B, 2C, and 3A, but also CYP2E1. However, contradictory results were obtained for CYP1A, while a decreased catalytic activity was observed for flavin-containing monooxygenases 1 and 3. The barbiturate had no effect on the chosen hydrolytic and conjugative DMEs. For the first time, we also measured the 26S proteasome activity, and the increase observed in PB-treated cattle would suggest this post-translational event might contribute to cattle DME regulation. Overall, this study increased the knowledge of cattle hepatic drug metabolism, and further confirmed the presence of species differences in DME expression and activity between cattle, humans, and rodents. This reinforced the need for an extensive characterization and understanding of comparative molecular mechanisms involved in expression, regulation, and function of DMEs.

## 1. Introduction

In mammals, xenobiotics can be metabolized by a variety of enzymes commonly referred to as drug-metabolizing enzymes (DMEs). These enzymatic reactions (“biotransformations”) are meant to convert xenobiotics in more hydrophilic derivatives, which ultimately are more easily excreted from the body. Together with uptake and efflux drug transporters, DMEs play an important role in the absorption, distribution, metabolism, and elimination (ADME) of xenobiotics [1,2,3,4]. In the past, biotransformation was invariably associated with deactivation or detoxification; however, this is not always the case, since in certain instances, through a process called “bioactivation”, DMEs may give rise to stable or unstable derivatives remarkably more (re)active than the parent compounds [5].

Drug-metabolizing enzymes are expressed in the liver and, albeit to a lesser extent, in many extrahepatic tissues, including circulating lymphocytes [6,7]. Furthermore, biotransformations are usually distinguished into phase I (oxidation, reduction, and hydrolysis) and phase II (conjugation with activated endogenous compounds) reactions. It is a shared opinion that members of the cytochrome P450 (CYP) enzyme superfamily (catalyzing oxidation reactions) are the most important phase I DMEs, which also include flavin monooxygenases (FMOs) and hydrolytic enzymes such as esterases (e.g., carboxylesterase, CES) and epoxide hydrolase (EH). The whole set of phase II DMEs consists of a certain number of enzyme superfamilies, including sulfotransferases (SULTs), UDP-glucuronosyltransferases (UGTs), and glutathione *S*-transferases (GSTs) [4,8,9,10].

Many factors may affect the overall biotransformation capacity, thus determining which pathway is involved in xenobiotic metabolism and the extent of these activating or detoxifying reactions. These factors are usually split into internal (e.g., species, strain/breed, gender, age, and physiopathological conditions) and external factors (e.g., diet, environment, induction/inhibition phenomena), as reviewed by Gibson, Skett, and Nebbia [2,5,11]. Obviously, this classification is purely arbitrary, and much interaction exists among these factors. In the present study, we focused and provided additional knowledge on two important factors known to affect DMEs expression, regulation, and function; i.e., species differences and enzyme induction [9,12,13,14,15].

The term induction denotes a dose-dependent increase in DME expression (gene/protein) and function (catalytic activity). Such a phenomenon, known for at least 60 years, is reported to influence the ADME of xenobiotics; among the possible consequences are an increased xenobiotic clearance, beneficial or harmful drug-drug interactions, and carcinogenicity, as well as an altered activity and disposition of relevant endogenous compounds (e.g., hormones). Cytochromes P450 are considered as the most important inducible DMEs; in particular, CYP1A1, 1A2, 2B6, 2C8, 2C9, 2C19, and 3A4 isoforms [4,15,16]. The mechanistic background of induction has been elucidated; most of the genes involved in drug metabolism and disposition are induced by specific xenobiotic-activated nuclear receptors (NRs); i.e., the aryl hydrocarbon receptor (AhR), the pregnane X receptor (PXR), the constitutive androstane receptor (CAR), peroxisome proliferator-activated receptors, and the 1α, 25-dihydroxy vitamin D3-activated vitamin D receptor. Drug-metabolizing enzymes’ transcriptional activation, and the resulting increased protein synthesis, follow the transactivation of xenobiotic-response elements present in the DNA of target genes. Additional NRs, such as the hepatocyte nuclear factor 4-α, the farnesoid X receptor, and the liver X receptor-α, play important roles in the metabolism of cholesterol and bile acids [4,9,16,17,18]. A notable exception is represented by the *CYP2E1* gene, the induction of which involves both transcriptional and post-transcriptional (protein stabilisation) mechanisms [15,19,20,21].

In humans and rodent species, a number of studies demonstrated that phase I and phase II DMEs were either induced or inhibited by several xenobiotics, including drugs, pesticides, food additives, industrial chemicals, natural compounds, environmental pollutants, and nutrients [9,15]. Basically, polycyclic aromatic hydrocarbons (e.g., β-naphthoflavone), barbiturates (e.g., phenobarbital, PB), glucocorticoids and polypeptide antibiotics (dexamethasone and rifampicin), fibrates (e.g., clofibrate), and short-chain alcohols (ethanol) are considered as prototypical CYP1A, 2B, 3A, 4A, and 2E1 inducers, respectively [4,22].

Phenobarbital is a widely used hypnotic and antiepileptic drug that causes pleiotropic effects in the liver, including an abnormal enlargement, hyperproliferation, and dysregulation of energetic homeostasis. Additionally, in humans and rodents, it is a prototypical inducer of the CYP2B, 2A, 2C, and 3A subfamilies; EH; some UGT, GST, and SULT isoforms; and a number of influx and efflux drug transporters [4,23,24]. Basically, PB upregulates CYP2B by activating CAR; however, regulatory cross-talks with PXR have been noticed; furthermore, the presence of alternative mechanisms for PB-mediated CAR activation (e.g., phosphorylation-mediated signal regulation) have also been hypothesised [22,25].

There is substantial literature affirming that PB and PB-like compounds; i.e., showing the same behaviour despite no evident structural relationship with PB or each other [26], induce human and rodent DMEs, and primarily CYP2B. However, species differences in the pattern of induction have been reported as well [9,15,27,28,29]. On a comparative basis, and especially looking at veterinary species, few data about the PB-dependent up-regulation of hepatic CYPs are actually available for pigs [30,31,32], sheep [33], rabbit [34], chicken [35,36], or dog [37,38,39]. However, contradictory in vitro results have been reported in the horse [40].

Cattle is an important food-producing species worldwide; however (and likewise to the abovementioned veterinary species), few data about the PB-mediated induction of DMEs are currently available. If we exclude the in vitro data from primary hepatocytes [41] and cocultures of hepatocyte and sinusoidal cell lines [42], the only available information is that published by Zancanella et al., in which the PB transcriptional effects on target DMEs, NRs, and drug transporters were measured in liver and extrahepatic tissues [24,43,44].

It is well established that differences, sometimes very consistent, are likely to exist between DME mRNA levels and coded enzyme activities; as a result, these post-translational variations, regardless of host and xenobiotic-related factors, may impact on the animal’s (individual) susceptibility to xenobiotics and target species risk assessment [45,46]. The purpose of the present study was to provide additional information on DMEs expression and catalytic activity in liver subcellular fractions from PB-induced cattle. Interestingly, we also investigated for the first time the ubiquitin-dependent 26S proteasome activity, in view of its role in protein (including CYPs) turnover [47,48,49]. This study was part of a larger project aimed at measuring transcriptional and post-translational effects of PB on cattle DMEs, NRs, and drug transporters [24,43,44]. Regarding liver post-translational results, only preliminary data have been published so far [50].

## 2. Results

### 2.1. Nuclear Receptors and CYP2B, CYP2C, and CYP3A mRNA Levels

**Figure 1:**
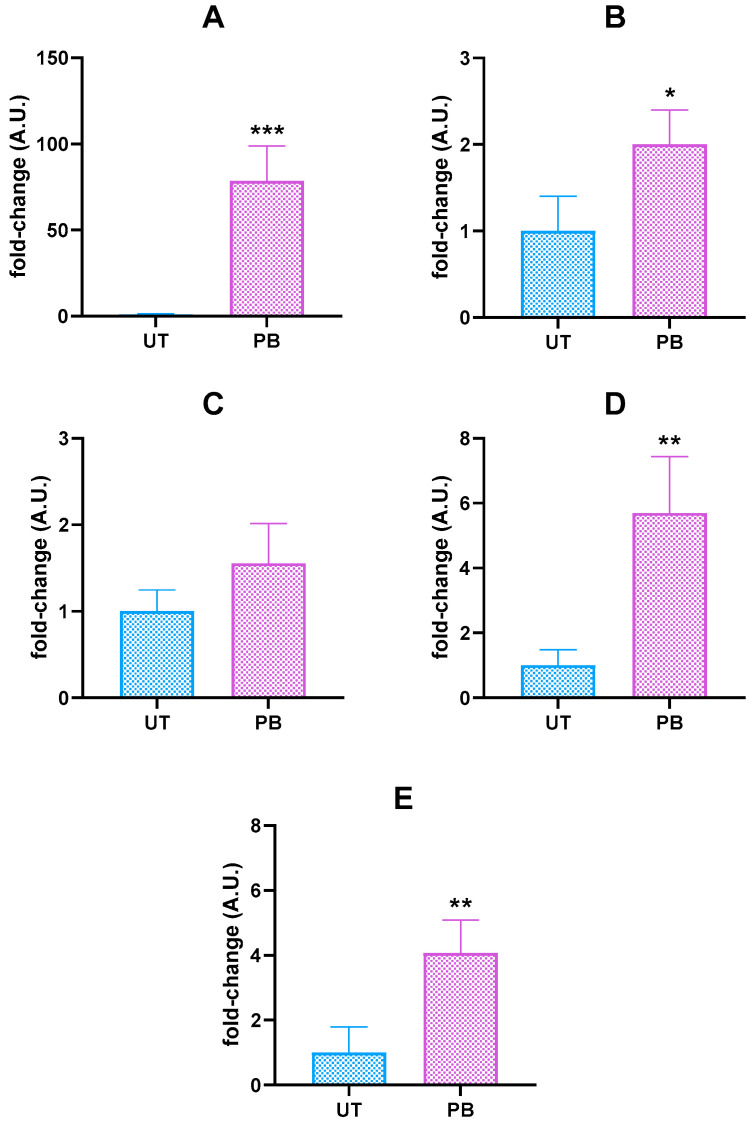
Hepatic *CYP2B22* (**A**), *3A* (**B**), *2C88* (**C**), *2C31* (**D**), and *2C42* (**E**) mRNA levels in untreated control (UT, *n* = 3) and phenobarbital-treated (PB, *n* = 4) cattle. Data (arithmetic means ± SD) are expressed as *n*-fold change (arbitrary units, A.U.) normalized to ΔΔCt mean value of β-actin (*ACTB*, the chosen internal control gene, ICG), to which an arbitrary value of 1 was assigned. * *p* < 0.05; ** *p* < 0.01; *** *p* < 0.001 (unpaired *t*-test).

To confirm the role of NRs in the PB-mediated induction of cattle DMEs, we measured *CAR*, *PXR*, and *RXRα* mRNA levels. No changes were noticed in the liver of PB-treated cattle (Figure S1). However, a remarkable upregulation of the *CYP2B22* gene (~78.5-fold higher than the UT value; *p* < 0.01; Figure 1) was observed in these same animals. Albeit to a lower extent, increasing mRNA levels were also noticed for *CYP2C31* (~5.7-fold; *p* < 0.01), *CYP2C42* (~4.1-fold; *p* < 0.01), and *CYP3A* (~2.0-fold; *p* < 0.05). In contrast, no differences were recorded for *CYP2C88* gene expression.

### 2.2. Haemoprotein Content, NADPH Cytochrome c (P450) and NADH Cytochrome b_5_ Reductase Activities, and Metyrapone Binding

Phenobarbital caused no changes in cytochrome *b*_5_ content and the NADPH cytochrome *c* (P450) reductase activity; however, it produced a significant (*p* < 0.01) increase in the total CYP amount (~2.0-fold vs. UT) and a ~64% inhibition (*p* < 0.001) of NADH cytochrome *b*_5_ reductase activity (Table S1). Furthermore, a significant increase (8.0-fold vs. UT; *p* < 0.01) of CYP binding to metyrapone was observed in liver microsomes isolated from PB-treated cattle.

### 2.3. Cytochromes-P450-Dependent Monooxygenases

#### 2.3.1. Cytochrome P450 2B22

**Figure 2:**
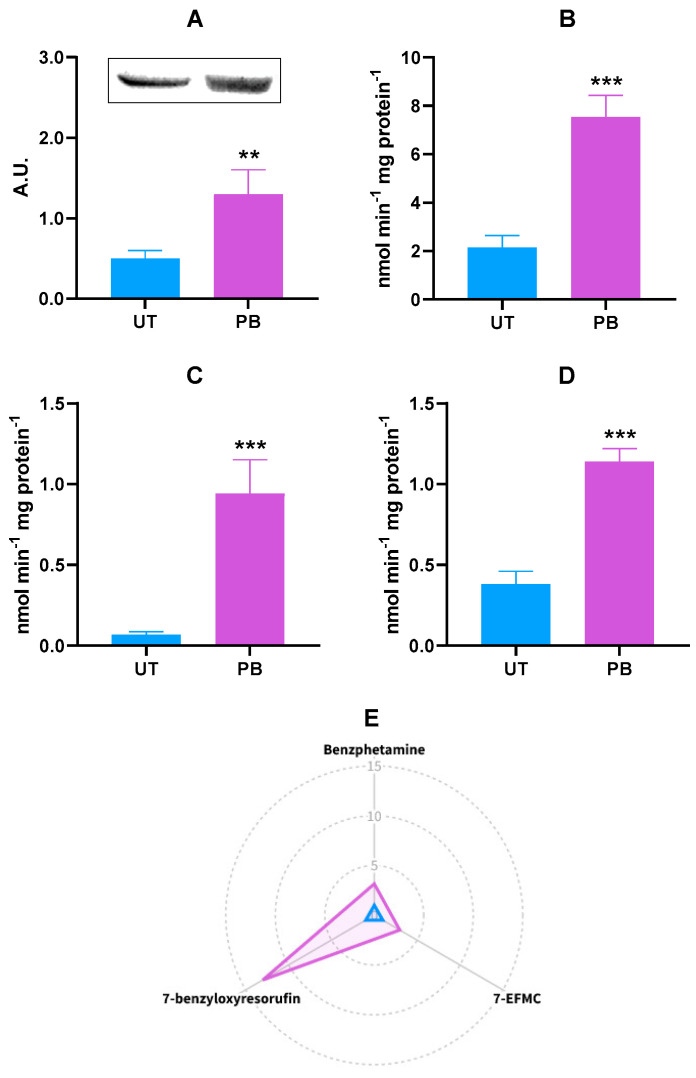
Hepatic CYP2B22 protein expression (**A**) and in vitro metabolism of CYP2B22 marker substrates benzphetamine (*N*-demethylation; (**B**)), 7-EFMC (*O*-demethylation; (**C**)), 7-benzyloxyresorufin (*O*-debenzylation; (**D**)) in untreated control (UT, *n* = 3) and phenobarbital-treated (PB, *n* = 4) cattle. In the radar plot (**E**), data are expressed in arbitrary units (A.U.), and a value of 1 was attributed to UT cattle. In the bar charts, data are expressed as arithmetic means ± SD. 7-EFMC: 7-ethoxy-4-trifluoromethylcoumarin. ** *p* < 0.01; *** *p* < 0.001 (unpaired *t*-test).

Similar to the *CYP2B22* mRNA levels, PB caused a significant, although less pronounced, upregulation of the CYP2B protein (~2.6-fold vs. UT; *p* < 0.01; Figure 2A). An unambiguous result was observed when measuring the in vitro metabolism of the selected CYP2B-dependent marker substrates benzphetamine, benzyloxyresorufin, and 7-EFMC; in fact, overall and significant inductions were observed (~3.5-, ~1.4-, and 3.0-fold vs. UT, respectively; *p* < 0.001; Figure 2B–D). Additionally, we also measured the extent of the *O*-depentylation of 7-pentoxyresorfin, another well-known CYP2B substrate. In our experimental conditions, such catalytic activity was undetectable in UT and barely quantifiable in PB-treated animals (6.16 ± 2.51 pmoles/min∙mg protein^−1^).

The radar plot is a form of radial graphing useful for the presentation of research outputs, especially whenever there are more independent variables with possibly different measurement scales [51]. To facilitate the readers’ understanding of results, a radar plot summarizing the in vitro metabolism of CYP2B22 marker substrates in both experimental groups is reported in Figure 2E.

#### 2.3.2. Cytochromes P450 2C88, CYP2C31, and CYP2C42

**Figure 3:**
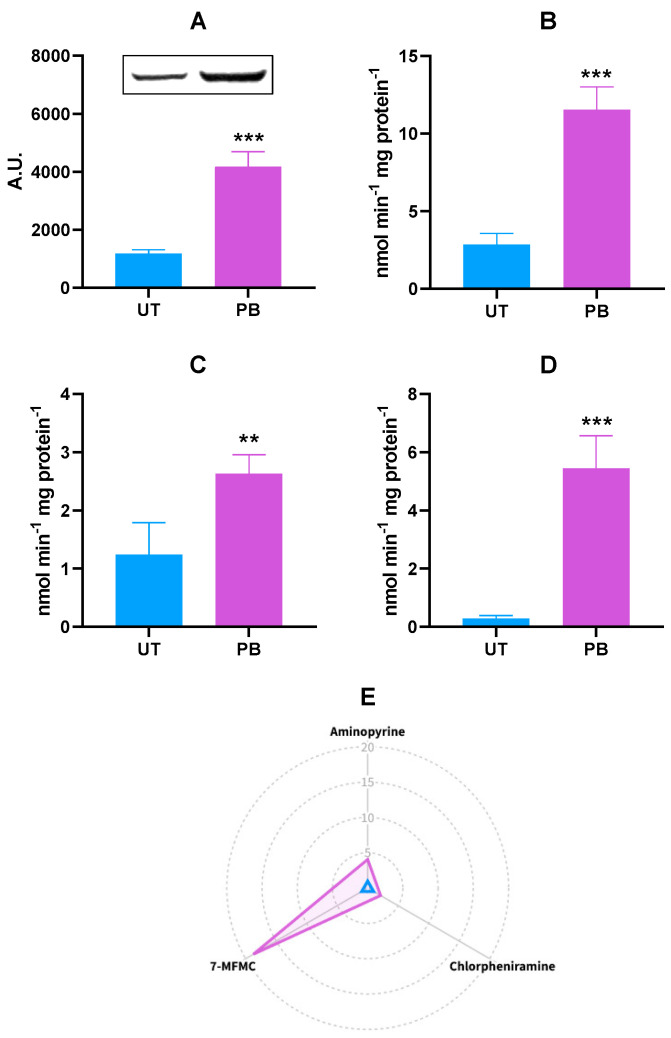
Hepatic CYP2C protein expression (**A**) and in vitro metabolism of CYP2C marker substrates aminopyrine (*N*-demethylation; (**B**)), chlorpheniramine (*N*-demethylation; (**C**)), and 7-MFMC (*O*-demethylation; (**D**)) in untreated control (UT, *n* = 3) and phenobarbital-treated (PB, *n* = 4) cattle. In the radar plot (**E**), data are expressed in arbitrary units (A.U.), and a value of 1 was attributed to UT cattle. In the bar charts, data are expressed as arithmetic means ± SD. 7-MFMC: 7-methoxy-4-trifluoromethylcoumarin. ** *p* < 0.01; *** *p* < 0.001 (unpaired *t*-test).

Although the PB transcriptional effects were assessed in genes coding for three different bovine CYP2C isoforms, a unique polyclonal antibody cross-reacting with human CYP2C8, CYP2C9, and CYP2C19 proteins was used to measure changes in bovine CYP2C protein levels resulting from PB administration. Overall, an increasing amount of a CYP2C cross-reacting protein was noticed (~3,5-fold vs. UT; *p* < 0.001; Figure 3A), thus partially confirming the *CYP2C31* and *CYP2C42* transcriptional results. In the present study, CYP2C-dependent catalytic activities were measured by using the broad substrates aminopyrine, chlorpheniramine, and 7-methoxy-4-trifluoromethylcoumarin (7-MFMC). The extent of each substrate’s demethylation was significantly enhanced by PB; in particular, ~4.0-fold for aminopyrine (*p* < 0.001), ~2.0-fold for chlorpheniramine (*p* < 0.01), and ~19.0-fold for 7-MFMC (*p* < 0.001; Figure 3B–D). The radar plot summarizing the in vitro metabolism of CYP2C marker substrates in both experimental groups is reported in Figure 3E.

#### 2.3.3. Cytochrome P450 3A

**Figure 4:**
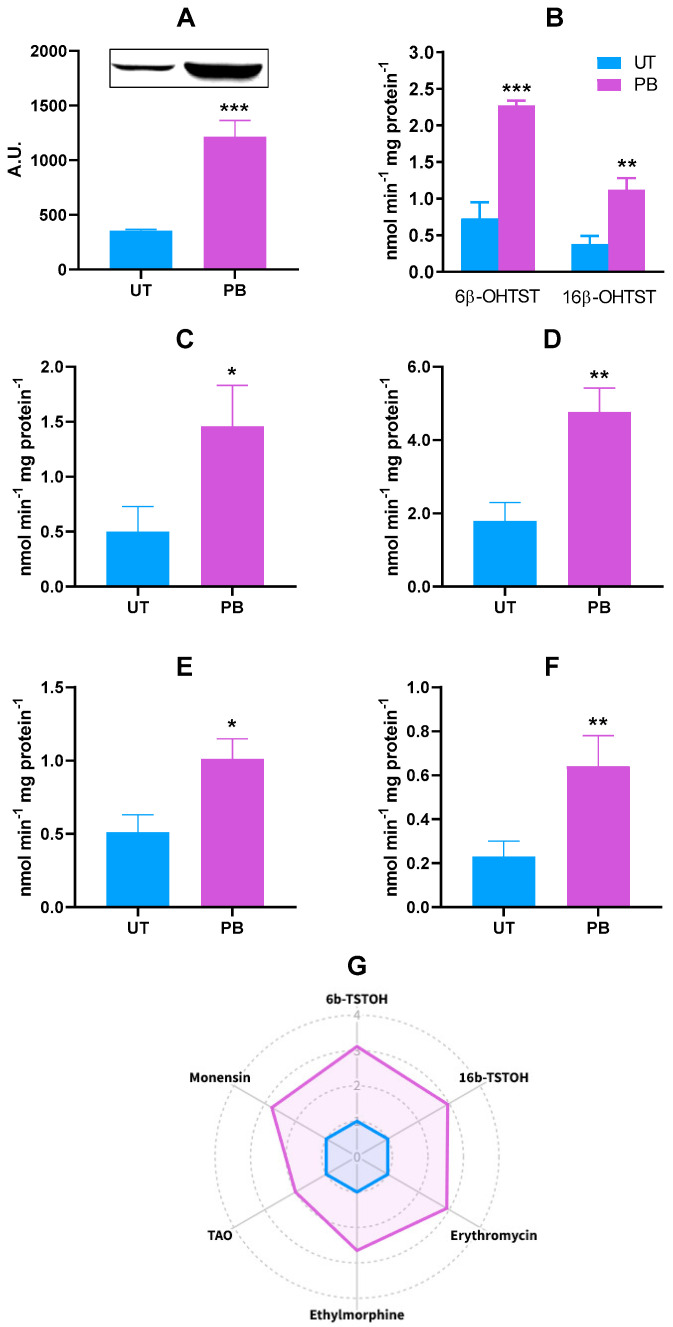
Hepatic CYP3A protein expression (**A**) and in vitro metabolism of CYP3A marker substrates TST (6β- and 16β-hydoxylation; (**B**)), erythromycin (*N*-demethylation; (**C**)), ethylmorphine (*N*-demethylation; (**D**)), TAO (*N*-demethylation; (**E**)), and monensin (*O*-demethylation; (**F**)) in untreated control (UT, *n* = 3) and phenobarbital-treated (PB, *n* = 4) cattle. In the radar plot (**G**), data are expressed in arbitrary units (A.U.), and a value of 1 was attributed to UT cattle. In the bar charts, data are expressed as arithmetic means ± SD. 6b-TSTOH: 6β-hydroxylated testosterone; 16b-TSTOH: 16β-hydroxylated testosterone; TAO: triacetyloleandomycin. * *p* < 0.05; ** *p* < 0.01; *** *p* < 0.001 (unpaired *t*-test).

When compared to *CYP2B22*, the pattern of *CYP3A* mRNA gene induction was a minor entity (Figure 1); despite this, the increased *CYP3A* gene transcription was confirmed at the protein level; specifically, a ~3.4-fold increase in CYP3A4 cross-reacting protein amounts was observed in microsomal proteins from PB-treated cattle (*p* < 0.001; Figure 4A). As to CYP3A-dependent catalytic activity, the best-known substrate testosterone (TST) was hydroxylated to a greater extent in PB-treated cattle than in UT, as shown by the higher detectable amounts of 6β- and 16β-hydroxylated derivatives; i.e., ~3.1-fold (*p* < 0.001) and ~2.9-fold (*p* < 0.01), respectively (Figure 4B). A similar behavior was also observed for the *N-*demethylation of other CYP3A substrates such as erythromycin, ethylmorphine, triacetyloleandomycin (TAO), and monensin, resulting in an increase of ~2.9- (*p* < 0.001), ~2.7- (*p* < 0.001), ~2.0- (*p* < 0.001), and ~2.8-fold (*p* < 0.001) in PB vs. UT microsomes, respectively (Figure 4C–F). The radar plot summarizing the in vitro metabolism of all the CYP3A marker substrates is reported in Figure 4G.

#### 2.3.4. Other CYP Monooxygenases (CYP1A, CYP2A, and CYP2E1)

Beyond CYP2B, 2C, and 3A, we also evaluated the effects of PB on members of the CYP1A, 2A, and 2E1 families. In our experimental conditions, controversial results were obtained for CYP1A. Significantly lower amounts of bovine microsomal proteins cross-reacting with the chosen anti-human CYP1A1/1A2 antibody were observed in PB-treated animals (−34.81% vs. UT; *p* < 0.05; Table S2); such a behavior was confirmed when using the specific CYP1A1 substrate 7-ethoxyresorufin (−34.12% vs. UT; *p* < 0.05). However, the rates of benzo[*a*]pyrene hydroxylation (another CYP1A1 probe substrate) and 7-methoxyresorufin *O*-dealkylation (a CYP1A2 preferential substrate) were significantly (*p* < 0.01) increased in PB-treated cattle (~0.8-fold and ~3.8-fold vs. UT, respectively). The 7-ethoxycoumarin is considered as a marker of both CYP1A1/1A2 and CYP1B1 [52]. Its pattern of dealkylation was consistent with benzo[*a*]pyrene and methoxyresorufin results (~2.0-fold vs. UT; *p* < 0.05).

In comparison with other human CYPs, the CYP2A family (consisting of three genes: *CYP2A6*, *CYP2A7*, and *CYP2A13*) plays a minor role in drug metabolism. The cytochrome P450 2A6 is the CYP2A isoform mostly expressed in the liver, and coumarin 7-hydroxylation is used as a marker reaction for CYP2A6-dependent catalytic activity [53]. No significant differences were observed in such a catalytic activity (Table S2). Hence, we did not measure the CYP2A6 apoprotein amount.

Regarding CYP2E1, an overall trend to induction was observed. Higher amounts of bovine proteins cross-reacting with an anti-rat CYP2E1 antibody were observed (~2.9-fold vs. UT), although such an increase was not statistically significant. On the other hand, significant increases in CYP2E1-dependent catalytic activities, namely the 4-hydroxylation of aniline (*p* < 0.05) and 4-nitrophenol (*p* < 0.01), were observed in PB-treated animals (~2.1-fold and ~2.9-fold vs. UT, respectively; Table S2).

### 2.4. Flavin-Containing Monooxygenases

Flavin-containing monooxygenases (FMOs) are expressed in bovine liver, and we measured the effects of PB in two of them: FMO1 and FMO3. No differences in cross-reacting FMO1 and FMO3 protein amounts were noticed between UT and PB-treated cattle. By contrast, the extent of *S*-oxidation of two common FMO substrates; i.e., ethylene thiourea (ETU) and methimazole (MTZ), was significantly (*p* < 0.01) lower in PB-treated animals (−34.5% and −39.8%, respectively; Figure S2).

### 2.5. Hydrolytic Enzymes

#### Carboxylesterases and EH

Concerning CES, we measured the effect of PB in three aromatic esters known to be CES probe substrates. The in vitro metabolism of two out of the three CES substrates was not affected by PB. The only exception was represented by α-naphtylacetate (ANA), for which a ~1.5-fold higher rate of hydrolysis (*p* < 0.05) was observed in PB-treated cattle (Table S3).

As to EH, we measured its catalytic activity by using *trans*-stilbene oxide (TSO); the barbiturate administration did not provoke changes in the enzyme activity (Table S3).

### 2.6. Conjugative Enzymes

#### 2.6.1. Glutathione (GSH) Content and GSTs

No significant changes in total hepatic GSH content and GST catalytic activities were ever recorded in PB-treated cattle, independently from the substrate used (Table 1).

#### 2.6.2. UDP-Glucuronosyltransferases

With regards to UGTs, conflicting results were obtained. Phenobarbital caused a significant inhibition of UGT activity toward chloramphenicol (−55.8% vs. UT, *p* < 0.01) and dexamethasone (−55.2%, *p* < 0.001). However, no changes were recorded for UGTs recognizing 1-napththol and *p-*nitrophenol as marker substrates (Table 1).

**Table 1:** Total glutathione (GSH) content, glutathione *S*-transferase (GST), and UDP-glucuronosyltransferase (UGT) in vitro metabolism in untreated control (UT, *n* = 3) and phenobarbital-treated (PB, *n* = 4) cattle.

Parameter	UT	PB
GSH ^∫^	1.99 ± 0.65	1.76 ± 0.26
CDNB GST ^#^	346.31 ± 30.32	312.56 ± 96.31
DCNB GST ^#^	334.87 ± 83.54	368.72 ± 18.56
ETA GST ^#^	5.10 ± 0.90	4.50 ± 0.59
Cumene hydroperoxide GST ^#^	366.00 ± 39.20	451.00 ± 118.00
1-Napththol UGT ^#^	13.40 ± 7.48	15.70 ± 1.74
*p-*Nitrophenol UGT ^#^	16.20 ± 8.90	23.20 ± 1.40
Chloramphenicol UGT ^#^	14.60 ± 1.70	6.45 ± 1.50 **
Dexamethasone UGT ^#^	11.25 ± 0.65	5.04 ± 1.17 ***

CDNB: 1-chloro-2.4-dinitrobenzene; DCNB: 3.4-dichloronitrobenzene; ETA: ethacrynic acid; Data are expressed as means ± SD. ** *p* < 0.05; *** *p* < 0.05 (unpaired *t*-test). ^∫^: μg/mg protein; ^#^: nmoles/min∙mg protein^−1^.

### 2.7. Proteasome Activity

**Figure 5:**
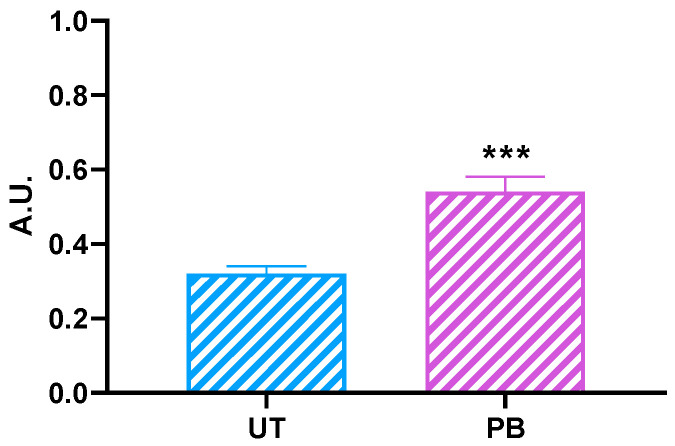
Proteasome chymotrypsin-like activity in untreated control (UT, *n* = 3) and phenobarbital-treated (PB, *n* = 4) cattle. Data are expressed as arithmetic means ± SD. A.U.: arbitrary unit. *** *p* < 0.001 (unpaired *t*-test).

Most unneeded or damaged cellular proteins are known to be degraded by the proteasome. Interestingly, the liver extracts from cattle administered with PB showed a significant (*p* < 0.001) increase in the 26S proteasome with a chymotrypsin-like activity (~1.68-fold vs. UT; Figure 5).

## 3. Discussion

Phenobarbital is a known prototypical inducer of DMEs; in humans and rodents, this barbiturate, which also causes pleiotropic effects in the liver, induces CYP2B, 2A, 2C, and 3A; EH; and some UGTs, GSTs, and SULTs [4,23]. However, if we considered such an evidence in a broader comparative context, remarkable species-related differences in the magnitude of response to PB have been reported [9,15,28,29,30,31,32,33,35,36,38,40]. Cattle is a worldwide important food-producing species, but few data about the PB-mediated induction of DMEs are available, and most refer to transcriptional changes in target DMEs, NRs, and drug transporters [41,42,43,44]. It is worth noting that differences may exist between DME mRNA levels and coded enzyme activities, ultimately resulting in post-translational variations that, regardless of host and xenobiotic-related factors, might impact on the animal’s (individual) susceptibility to xenobiotics and risk assessment in the target species [45,46]. The present study aimed at providing additional information on hepatic DME expression and catalytic activity in PB-administered cattle. Only preliminary data about liver have been published so far [50]. Additionally, for the first time, we also measured the activity of the cattle hepatic ubiquitin-dependent 26S proteasome, an ATP-dependent proteolytic machine involved in CYP turnover [54].

### 3.1. Nuclear Receptors and CYP2B, CYP2C, and CYP3A mRNA Levels

The assessment of PB’s effects on target gene expression was not the primary objective of the present study. Indeed, data about PB transcriptional effects on a number of bovine hepatic and extrahepatic NRs, DMEs, and drug transporters have been previously published [24,43,44]. Nevertheless, we thought it would be useful to confirm the role of NRs primarily involved in PB-mediated transactivation of target CYPs (i.e., *CAR*, *PXR*, and *RXRα*; and *CYP2B*, *CYP2C*, and *CYP3A*) [4,15,55,56]. In our study, PB did not cause changes in liver NR mRNA levels; however, a significant induction was observed in target CYPs, except for *CYP2C88* (*CYP2B22* >> *CYP2C31* *≃ CYP2C42* > *CYP3A*). The transcription factors CAR and PXR and the common heterodimerizing partner RXRα transcriptionally mediated the induction of CYP2B, CYP2C, and CYP3A in human hepatocytes in the presence of PB [4]. Moreover, CAR and PXR showed a relatively high degree of similarity; hence, there was a considerable cross-talk between these NRs. Phenobarbital is considered as an activator of both NRs, although CAR constitutive expression has been hypothesized to contribute more extensively to the magnitude of *CYP2B* induction [57,58]. Despite all this, present and apparently contradictory NR transcriptional results are not surprising. Species differences in ligand specificity (i.e., rifampicin and pregnenolone-16α-carbonitrile vs. PXR) exist between humans and rodents [59], ultimately resulting in differential transcriptional regulation (hence, in the pattern of induction); on the other hand, human and pig primary hepatocytes showed similar responses in the presence of prototypical CAR and PXR activators, including PB [60]. Variations in NR ligand-binding domain sequences, differences in CAR/PXR-dependent transactivation of target genes (e.g., *CYP2B*), as well as dose- and time-dependent differential responses, have been called into question to explain species differences in CYPs response to PB, PB-like compounds, and to a wider extent, prototypical CYP inducers [58,61,62,63]. With regard to cattle, the abovementioned hypotheses would be supported by results obtained in previously published in vitro/in vivo studies [44,64,65,66]. Additionally, the presence of alternative noncanonical NR (CAR) activation mechanisms (e.g., a PB-dependent increased phosphorylation-mediated signal regulation) have also been recently hypothesised [25]. Taken together, previous and present results suggest that further basic and applied molecular studies are needed to understand the basic (ligand-mediated) or alternative (e.g., ligand-dependent or independent phosphorylation-mediated signaling) regulatory mechanisms by which PB transactivates cattle CAR/PXR, in any case resulting in an increase in target *CYPs* gene transcription.

### 3.2. Haemoprotein Content, Members of the CYP Catalytic Cycle, and Metyrapone Binding

It is well known that measuring the total CYP content gives an indication of the overall xenobiotic metabolism capacity; nevertheless, such a parameter is nonselective, as it is impossible to identify which CYP isoform is specifically involved in the oxidative metabolism of a given xenobiotic. This bias can be solved by using either selective CYP inducers/inhibitors or CYP isoform-specific probes, or both approaches [67,68], as we did in the present study. Even though PB elicits pleiotropic effects in the exposed organism, it is known that it increases the total CYP content in the liver [33,67,69,70,71,72,73,74,75,76]. A similar behavior was observed in our experimental conditions.

The xenobiotic oxidation by CYPs involves a complex catalytic cycle in which cytochromes *b*_5_*,* as well as the cytochrome *c* and CYP reductases, play an important role [77,78]. While species differences in total CYP content have already been reported, much less is known about the constitutive expression of the other members of the CYP catalytic cycle [10]. In theory, it would be justifiable to hypothesize that these proteins answer unambiguously to PB administration, but this was not the case. In our experimental conditions, the only variation we found was a significant decrease in NADH cytochrome *b*_5_ reductase activity. Since the 1970s, the effects of PB on this reductase have been the subject of investigations [79,80], and an inhibition has already been recorded in rats and rhesus monkeys administered with the barbiturate [74,81,82]. However, contradictory results were obtained for the other members of CYP catalytic cycle. Although in our case, the cytochrome *b*_5_ amount was in line with what previously observed in monkeys and rats [70,74,81], it is difficult to explain the lack of effect on NADPH cytochrome *c* (P450) reductase activity [69,75,76,81,82,83]. Apart from chemicophysical factors (e.g., pH, heat stability, and the presence of glycerol), both cattle and sheep liver reductase was involved in the *N*-demethylation of benzphetamine [84], a CYP2B substrate that was induced in our experimental conditions (Figure 2). Moreover, PB induced the reductase throughout the rat liver lobule, while differential effects were observed with two other prototypical CYP inducers: pregnenolone 16a-carbonitrile and 3-methylcholantrene [85]. Hence, further and species-specific biomolecular studies are needed to clarify these contradictory results.

Metyrapone is a potent inhibitor of adrenal CYP11β, which inhibits steroidogenesis [86,87], and since the 1960s, it has been used for the treatment, either alone or in combination with mitotane, of Cushing’s syndrome [87,88]. In the field of xenobiotic metabolism, it acts as a CYP nonspecific type II ligand [88]; hence, it is used as a general CYP inhibitor, but particularly of CYP3A4 [86,89,90,91]. Interestingly, metyrapone has been successfully used to assess the PB-dependent induction of CYPs and mostly CYP2B isoforms [92]; moreover, the in vitro contemporary measurement of the TAO metabolite complex and metyrapone binding to reduced CYP allowed a selective spectroscopic quantitation of CYP3A and 2B isozymes, respectively [93]. The increasing extent of CYP-metyrapone binding in liver microsomes from PB-treated cattle is consistent with the enhanced response of bovine CYP2B22 to the barbiturate, as previously observed in other mammalian species.

### 3.3. Cytochromes-P450-Dependent Monooxygenases

Before addressing the post-translational effects of PB on cattle DMEs, we would like to highlight that the choice of a specific substrate characterizing the catalytic activity of a given DME still represents a potential bias in the methodological approach to xenobiotic metabolism studies in veterinary species. Despite a specific demand, and the consequent efforts put in place in the past decade, a full characterization of species-specific DME catalytic activities, including the identification of the best substrate marker, is still far from being completed. With few exceptions, we still use substrates derived from human and rodent databases, the kinetic parameters of which (i.e., K_m_ and V_max_) in the target species are often unknown or only have been occasionally measured. This makes it difficult to extrapolate xenobiotic metabolism data across different species, as well as to reach a correct and exhaustive understanding of the constitutive expression and catalytic activity of DMEs [14]. Since the 1970s, a common approach to overcome this pitfall has been the contemporary measurement of each DME’s catalytic activity by using more than one marker substrate. This approach was also adopted in studies on cattle xenobiotic metabolism, and especially in comparative studies [8,10,94,95,96,97,98,99,100,101,102,103]. Some of these studies represented the starting point for the evaluation of cattle DME activities. However, it should be underlined that some enzyme-substrate kinetic studies have already been published for bovines [97,104,105,106,107,108].

There is substantial literature on the inducibility of human and rodent CYP2B enzymes by PB [9,15,69,109,110]. In our experimental conditions, cattle did not behave differently: in complete agreement with *CYP2B22* mRNA levels, PB caused a significant, albeit less consistent, increase in the CYP2B protein. Furthermore, the barbiturate provoked an explicit and significant increase in the in vitro metabolism of the chosen CYP2B22-dependent substrates; i.e., benzphetamine [10,100,102], benzyloxyresorufin [10,94,96,101], and 7-EFMC [94]. Some of the present results corroborated those previously observed in other veterinary species treated with PB, such as sheep [33], beagle dog [39,111], pig [30], and rabbit [34]. However, PB (100 μM) did not induce the CYP2B-depedent *O*-demethylation of 7-EFMC in horse primary hepatocytes [40]. Another known CYP2B substrate is 7-pentoxyresorufin, which normally undergoes *O*-dealkylation in humans [109], rodents [112,113], cattle, and other veterinary species [94,96,97,101]. In basal conditions, this catalytic activity, even in cattle, was either undetectable or very low (few pmoles/min∙mg protein^−1^) [97,101,109,112,113]. In our experimental conditions, it was undetectable in UT; on the other hand, it was measurable in liver microsomes from PB-treated cattle. Collectively, our data unambiguously confirmed the cattle CYP2B22’s whole responsiveness (RNA-protein-enzyme activity) to PB when used as a prototypical DME inducer. Moreover, the present results suggested benzphetamine as the most sensitive CYP2B substrate in cattle, although specific enzyme kinetic studies are needed to corroborate such evidence.

In humans, the CYP2C subfamily metabolizes some commonly prescribed drugs (e.g., phenytoin, diclofenac, omeprazole, celecoxib, clopidogrel, and paclitaxel); however, despite the high homology in DNA and protein sequences (> 82%), each CYP2C member is unique in substrate specificity and its role in drug metabolism [56,114]. Overall, PB induces the CYP2C subfamily in humans and rodents, but species-related differences in responsiveness have also been noticed (e.g., only CYP2C29 and 2C37 are induced by PB in the rat) [56,115,116,117]. Overall, it is believed that the CYP2C subfamily is expressed in veterinary species [14,118]. However, the situation in cattle is more complicated. In 2010, a phylogenetic analysis led to the proposition of a new nomenclature for CYP2C and 3A, which mirrored the true evolutionary relationships of bovine CYPs [119]. Additionally, the new updated version of the bovine genome database (http://bovinegenome.org, accessed on 19 January 2022) brought about substantial changes to the abovementioned nomenclature. Finally, scarce information is available about the substrates helpful in assessing the specific role of each member of this CYP subfamily in cattle drug metabolism, and isoform-specific antibodies are still not available. In our study, a unique polyclonal antibody raised against human CYP2C8, CYP2C9, and CYP2C19 proteins (CYP2C18 is expressed at the mRNA but not at the protein level in the liver) [56] was used to detect bovine CYP2C proteins. An increasing amount of a cross-reacting CYP2C protein was observed in the liver of PB-treated cattle, thus partially confirming transcriptional results (i.e., *CYP2C31* and *CYP2C42*). Bovine CYP2C-dependent catalytic activities were evaluated by using the broad substrates aminopyrine [95,120,121], chlorpheniramine [122,123], and 7-MFMC [94,101,123]. Phenobarbital significantly increased the in vitro metabolism (*N-*demethylation) of the abovementioned substrates, and especially of 7-MFMC. These results were quite clear, and at least partly confirmed those obtained in canines [111] and pigs [30]; nevertheless, they should be considered with caution, in light of the shortcomings in bovine CYP2C expression, regulation, and substrate specificity mentioned above.

The cytochrome P450 3A subfamily represents the main CYP isoform in human liver [114]. Although the magnitude of CYP3A biological response is lower than CYP2B for a number of reasons; e.g., different inducing potencies and the molecular mechanisms involved, PB induced CYP3A in humans and rodents [124,125,126,127]. In bovines, three *CYP3A* genes have been identified: *CYP3A28* (the human *CYP3A4* orthologue), *CYP3A38* (orthologue of human *CYP3A5*), and *CYP3A48* (corresponding to a bovine *CYP3A4* “nifedipine oxidase”). Actually, *CYP3A38* is believed to be the most abundant *CYP3A* isoform in bovine liver, followed by *CYP3A48*; moreover, PB transcriptionally induces these *CYP3As* (*CYP3A38* > *CYP3A28* > *CYP3A48*) [43]. In the present study, increasing amounts of CYP3A mRNA and protein, though of a lesser magnitude compared to those recorded for CYP2B22, were observed in bovines administered with PB. A certain number of CYP3A substrates were used to assess cattle CYP3A-dependent enzyme activities, and PB provoked an overall and significant increase in their in vitro metabolism. The present results confirmed those previously obtained in sheep [33], canines [111], and pigs [30].

Despite there is a wealth of information describing the univocal response of CYP2B, 2C, and 3A to PB (i.e., induction), less clear or sometimes contradictory results have been published about the effects of PB on other CYP monooxygenases. Among these, PB seems to upregulate the CYP1A, 2A, and 2E1 subfamilies [23,124,128,129].

The human CYP1A subfamily comprises two highly conserved genes: *CYP1A1* and *CYP1A2.* The former is an extrahepatic DME, whilst CYP1A2 is highly expressed in the liver [114]. At present, no data on the constitutive expression of CYP1A2 in bovine liver (e.g., resulting from quantitative proteomics, immunoblotting with species-specific CYP1A1 and CYP1A2 antibodies) are actually available. In our study, cattle CYP1A-dependent catalytic activities were assayed using known substrates [10,94,95,96,97,100,101,103,130]. Overall, we obtained contradictory results; whether the CYP1A protein amount and 7-ethoxyresorufin *O*-deethylase (EROD) activity were lowered in PB-treated cattle, statistically significant increases were instead noticed in the *O*-demethylation of 7-methoxyresorufin and 7-ethoxycoumarin, as well as in the hydroxylation of benzo[a]pyrene. In humans, the catalytic activities of CYP1 enzymes are overlapping, although the prototypical biotransformations catalyzed by CYP1A2 include EROD [114]. Regarding cattle, further studies are needed to identify probe substrates to measure CYP1A2- and CYP1A1-dependent catalytic activity. In veterinary species, few and contrasting results of the effects of PB on CYP1A have been published only in canines [39,73,131]. However, both human and rodent CYP1A1/1A2 were induced by PB [132,133,134]. In this respect, it has been hypothesised that PB-dependent induction of CYP1A occurs through transcriptional and post-transcriptional mechanisms. Specifically, studies in mouse hepatoma cells proved PB to be a weak ligand of AhR, as well as an inducer of CYP1A1 and benzo[a]pyrene hydroxylase activity [133]; on the contrary, CYP1A2 was regulated through molecular mechanisms independent from AhR [135]. Finally, a binding site for the CAR/RXRα heterodimer (an ER8 motif) has been identified in the proximal promoter of human *CYP1A1*. This would suggest that PB might transactivate *CYP1A1* (and possibly *CYP1A2*) also through the activation of CAR [134]. Although our results would partially confirm this evidence, additional molecular studies are needed to clarify the regulation of the cattle *CYP1A* gene family; in particular, further research should focus on the possible presence of differential NR-dependent (i.e., AhR- or CAR-mediated) transcriptional mechanisms of gene regulation or, alternatively, post-transcriptional events.

In addition to humans, the CYP2A subfamily has been characterized in rodents, rabbit, pigs, and cattle [114,136]. In humans, mice, and pigs, PB induced CYP2A6 through the involvement of CAR [114,137]. The 7-hydroxylation of coumarin was a selective marker activity for CYP2A6 in humans, pigs [52,114,138], and cattle, although with some differences in enzyme kinetics [91]. In our experimental conditions, we did not observe significant differences in coumarin hydroxylation between UT and PB-treated cattle. Marked species differences in CYP2A catalytic activities, in response to inducers (including PB), and possibly in regulation can be offered as a justification of the present results [136,139,140].

The cytochrome P450 2E1 is constitutively expressed in humans, rats, mice, and most veterinary species [10,94,95,96,97,101,105,141,142]. Interestingly, CYP2E1 regulation involves complex and different mechanisms; i.e., gene transcriptional activation, mRNA and CYP2E1 protein stabilization, and a possible increased efficiency of mRNA translation as well [114,128,142]. Overall, chlorzoxazone is still considered as the ideal substrate marker for CYP2E1 catalytic activity, not only in humans, but also in several mammalian species, including cattle [97,105]. However, other probes have also been successfully used to characterize the CYP2E1 catalytic activity [10,95,96,130,143,144,145]. As a whole, we can say that PB is likely to induce CYP2E1, even though contradictory results have been published [128,140,141,143,146,147,148]. In our study, an increasing oxidative capacity toward the selected CYP2E1 probes aniline and 4-aminophenol was observed in liver microsomes isolated from PB-treated cattle; however, such a behavior was only partially confirmed at the protein level. Because of the complexity of molecular mechanisms affecting CYP2E1 regulation, it is our opinion that additional molecular studies might be required to clarify CYP2E1 expression and regulation, and consequently, its biological function.

### 3.4. Flavin-Containing Monooxygenases

At present, five active FMOs have been observed in humans, but they were not inducible by classical CYP inducers, including PB [149,150]. Similar to humans, FMOs are constitutively expressed in cattle liver; notably, they participate together with CYP1A in the oxidative metabolism of an important class of antiparasitic agents; i.e., the benzimidazole anthelmintics [151,152]. In humans and rodents, both MTZ and ETU, an ethylenebisdithiocarbamate fungicide derivative also formed in cattle [153], are oxidized by FMOs [154,155,156]. The transcriptional regulation of FMOs, which involves NR ligand binding and interaction with DNA, has not been as widely studied as other DMEs. Similarly, the possible post-transcriptional regulation of FMOs has been poorly investigated [157]. In the present study, PB did not affect the expression of FMO1 and 3 protein amounts, thus confirming that mammalian FMOs were not inducible by prototypical inducers [150,158]. However, the FMO-dependent *S*-oxidation of MTZ and ETU were significantly reduced in PB-treated cattle. A number of representative FMO substrates have been identified [159], and some of them have also been proved to be competitive inhibitors of these monooxygenases, thus leading to a decreased FMO catalytic activity. Methimazole is one of these [152,160]. However, ETU has been shown to bind covalently microsomal proteins, and species and gender differences have been noticed as well [154,156]. We hypothesized that MTZ and ETU inhibitory concentrations of cattle FMOs might have been used, and PB induction emphasized the reduction in FMO-dependent catalytic activity. Clearly, confirmatory studies are needed to corroborate such a hypothesis.

### 3.5. Hydrolytic Enzymes

A number of hydrolases have been identified and proved to be involved in xenobiotic metabolism and toxicity, including bioactivation reactions. Carboxylesterases, EH, and paraoxonase are the most important drug-metabolizing hydrolases [161,162,163].

Human CES consists of five isoforms, and CES1 and CES2 are those mostly involved in xenobiotic hydrolysis. Carboxylesterase 1 is predominantly expressed in the liver, while CES2 is expressed at higher concentrations in the gastrointestinal tract [164]. The substrates of both CES isoforms commonly possess a small acyl group in their chemical structures. Hence, the CES substrate specificity may be predicted by looking at the size of this acyl group [162]. Regarding PB, it moderately induced CES1 and CES2 in human hepatocytes [165], while age differences in PB inducibility were observed in mice [166]. To our knowledge, few papers have measured the CES catalytic activity in veterinary species, including cattle [8,99]. In our study, we used three different probes due to marked differences in CES substrate preferences [167]. Significant changes (~1.5-fold higher than in the untreated control) were only noticed when using ANA as a substrate. The present results showed the absence of a relationship between ANA and IPA/PNP hydrolysis rates. Therefore, we hypothesized the presence of species-specific variation in CES isozyme abundancies and differences in the substrate preference, as already supposed in fish [168].

Epoxide hydrolases are ubiquitous and evolutionarily highly conserved DMEs that catalyze the opening of the epoxide ring of xenobiotics and endogenous compounds by adding water, thus generating a dihydrodiol, a reaction product that is more easily excreted [163,169]. Mammalian EHs consist of five members, but only two of them are of ultimate significance; i.e., the microsomal EH (EPHX1) and the soluble EH (EPHX2) [163,170]. Human EPHX1 is responsible for the metabolism of xenobiotic epoxides [163]; e.g., the aflatoxin B1 exo-8,9-epoxide detoxification and the metabolic activation (together with CYP1A1) of the human carcinogen benzo[*a*]pyrene [171,172]. Despite being involved in xenobiotic epoxide metabolism, EPHX2 mostly metabolizes epoxides derived from fatty acids [163]. Conflicting opinions exist on EPHX1 substrates. Trans-disubstituted (such as TSO) and trisubstituted epoxides are considered as poor substrates for EPHX1 [169]; however, EPHX1 and EPHX2 showed partly redundant functions, including an overlap in substrate selectivity and identical biological activity [163]. In the present study, we used TSO as a substrate essentially for two reasons. First, it is considered as a “PB-like” compound, because it induces hepatic CYP2B1/2 and other DMEs, including EPHX1 as well [163,170]. Furthermore, TSO was occasionally used to measure the EH basal activity in ruminants, including cattle [96,98,100,102,103]. Regarding EH inducibility by PB, contradictory results have been published in the past, and such an issue is still controversial [163,169,170,173,174]. It is worth noting that no data about EH responsiveness to PB are available for cattle or other veterinary species. In our experimental conditions, PB showed no effects on cattle EH enzyme activity, but it was evident that further investigations are warranted to clarify the role of EH, and particularly of EPHX1, in comparative drug metabolism and cell homeostasis.

### 3.6. Conjugative Enzymes

Basically, phase II DMEs are enzyme superfamilies, consisting of families and subfamilies of genes encoding isoforms with different tissue expression and regulation, substrate specificities, and pattern of induction/inhibition by xenobiotics. Human UGTs, GSTs and SULTs are the main conjugative DMEs, participating in the metabolism of drugs most commonly used in therapy [4,175,176]. At present, limited information is available about SULT expression, regulation and catalytic activity [177], but phenol and 2-naphthol have already been used as probe substrates in ruminants [98,100,102]. The sulfotransferase 2A1-*like* is the predominant SULT isoform in bovine liver, but PB exposure did not affect its mRNA levels [44]. Therefore, we did not investigate the effect of PB oral administration on SULT enzyme activities, but we focused on the other two major conjugating DMEs, i.e., UGTs and GSTs.

#### 3.6.1. Glutathione Content and GSTs

Glutathione, GST isoforms, and glutathione peroxidase are commonly considered as the hinge of the cellular antioxidant response [178,179,180,181]. Regarding GSH and GSTs, they inactivate a huge number of xenobiotics (e.g., carcinogenic alkylating agents), natural toxins, and relevant endogenous compounds. In most species, the common reaction involves either the transfer of a thiol group (reduction) or the xenobiotic direct conjugation with GSH itself, via a thioether linkage. Nevertheless, human GSH and GSTs are involved in many other pleiotropic functions [179,180,181]. Similar to humans, the major amount of cattle GSH is synthetized in the liver and exported in blood and bile by transporters. Therefore, a depletion in hepatic GSH; e.g., due to increased xenobiotic metabolism or oxidative stress, may result in impaired GSH supply and altered animal homeostasis [182]. Despite the pivotal role of GSH played in detoxification pathways, few papers on the role of GSH in bovine hepatic xenobiotic metabolism have been published [98]. Concerning the possible modulatory effect of PB on the total GSH content, contradictory results have been published in rodents: while significantly increasing levels of hepatic GSH have been noticed in rats pretreated with PB or following an acute exposure to the barbiturate [75,183], in other experiments in rats and mice, PB showed no effect on hepatic GSH content [184,185]. Our results confirmed this second hypothesis, as no changes in total hepatic GSH content were noticed in PB-treated cattle.

Much more is known about GSTs, the third most important conjugative enzyme that participates in the metabolism of clinically used drugs [175]. The glutathione *S*-transferases superfamily encompasses three evolutionarily distinct gene families; i.e., cytosolic, mitochondrial, and microsomal GSTs [181,186,187] In humans, eight GST subfamilies have been identified [181]. These conjugative DMEs possess pleiotropic functions, but they are mostly involved in the detoxification of both endogenous and xenobiotic electrophilic derivatives (e.g., epoxides), more often than not resulting from a bioactivation process. A certain number of carcinogens, drugs, natural toxins, and products of oxidative stress are usually detoxified by GSTs [175,181,187]. A certain number of GST substrates have been identified, but 1-chloro-2.4-dinitrobenzene (CDNB) is generally considered as the “universal” one [8,175,186,187].

Regarding cattle, basal GST catalytic activities have already been measured by using the most common human and rodent marker substrates, and interesting tissue- and species differences in the conjugation efficiency have been observed [8,96,98,100,101,102,103,188,189,190,191]. Interestingly, the available data suggest the *GSTA1-like* gene as the foremost *GST* isoform in bovine liver compared to extrahepatic tissues, even though similar amounts of target mRNA have also been detected in kidney, lungs, and testes [44,192,193]. In humans and rodents, there is a substantial bibliography on GST inducibility by PB, even though isoform differences in the pattern of induction, as well as in transferase activity toward specific substrates, have been observed [2,23,173,187,194,195,196,197]. In our experimental conditions, PB did not increase GST activities independently from the substrate we used. To the best of our knowledge, this is the first report on the possible PB-dependent in vivo modulation of bovine hepatic GST catalytic activities; the present results partially disagreed with those published by Zancanella et al. (2012), in which increasing amounts of the *GSTA1-like* gene and protein were noticed [44]. It seems obvious that the role played by cattle GSTs in endobiotic and xenobiotic metabolism needs to be elucidated more in depth, at first by implementing the biomolecular mechanisms involved in their expression, regulation, and biological activity, in light of the outstanding role played by this conjugative DME in living organisms, including bovines.

#### 3.6.2. UDP-Glucuronosyltransferases

UDP-glucosyltransferases are a superfamily of conjugating enzymes that play an important role in the metabolism of endogenous and exogenous compounds. Mammalian UGTs essentially comprise two enzyme families (UGT1 and UGT2), which can be in turn divided into three subfamilies (UGT1A, UGT2A, and UGT2B) [198,199,200]. In humans, UGTs are the major phase II DMEs, since more than 20% of clinically used drugs undergo glucuronidation [175,201]. Humans and laboratory species (including dogs) express different UGTs; moreover, they show different tissue distribution when compared with human orthologues. As a whole, UGTs recognize a broad and often overlapping set of exogenous and endogenous substrates [13,200,202,203]. Studies measuring UGT catalytic activities in cattle and other veterinary species have already been published [8,96,100,101,102]. In most cases, the two human UGT1A probe substrates 1-napththol and *p-*nitrophenol were used, and cattle showed a relatively low rate of glucuronidation [8]. Several laboratories proved the PB-dependent induction of UGTs, and particularly of UGT2B1 and 1A1 isoforms [198,201,202,204]. However, species differences in the magnitude of induction between humans and rodents have been recorded as well [28,201]. To our knowledge, data on hepatic UGT activities in cattle exposed to PB have never been published so far. The present results seemed to contradict what was mentioned above; the barbiturate halved the UGT-dependent conjugation of chloramphenicol and dexamethasone, whereas no changes were noticed in the pattern of glucuronidation of both 1-napththol and *p-*nitrophenol. In cattle liver, the *UGT1A1-like* is the predominant *UGT* coding gene; moreover, *UGT1A1-like* mRNA and coded protein amount were significantly increased by PB [44]. Constitutive and inducible UGTs have been shown to be primarily regulated transcriptionally by NRs, in primis in the members of the hepatocyte nuclear factor family of transcription factors, as well as in tissue-specific transcription factors [199,200,205,206]. Furthermore, additional mechanisms are likely to contribute to UGT basal activities and induction/repression phenomena; e.g., DNA methylation and histone modifications, activation and repression by diverse transcription factors, mRNA stability and/or translation by microRNAs, and post-translational modifications [199]. Bovines showed a differential transcriptional response to human and rodent CAR and PXR agonists and inverse agonists [66]. These NRs contribute to human *UGT1A* gene regulation, the enzyme activity of which can be measured by using 1-napththol and *p-*nitrophenol [205,207]. The present results suggested caution in any attempt to compare bovine glucuronidation as a whole (e.g., expression, regulation, and biological activity) with data available for human, rodents, and other veterinary species.

### 3.7. Proteasome Activity

The 26S proteasome is a gigantic multicatalytic, ATP-dependent protease complex that serves as the degrading arm of the ubiquitin system; the latter represents the foremost pathway for the regulated degradation of nuclear, cytosolic, and membrane proteins [208,209,210,211,212,213]. Regarding DMEs, there is enough information about CYPs; these hemoproteins undergo proteolytic turnover through a process involving either an ubiquitin-dependent 26S proteasomal degradation or an autophagic-lysosomal degradation [47,214]; moreover, CYPs incur phosphorylation after functional inactivation, and such an event should be viewed as a necessary “marking” for degradation [208,214,215]. Human and rat CYP3A and 2E1 are turned over via phosphorylation and 26S proteasomal degradation, whereas CYP2B1 and 2C11 are largely degraded by the autophagic lysosomes [214,215,216,217,218]. However, the reasons for such a heterogeneity and differential proteolytic targeting remain poorly characterized [54]. To our knowledge, no data are actually available on the involvement of the 26S proteasome in the turnover of the other DMEs subjected to investigation in the present study; e.g., GSTs and UGTs. Overall, little information is currently available on proteasomes’ biological functions in cattle and other domestic animals [210,211]. Interestingly, increasing evidence suggests that the 26S proteasome complex may indirectly contribute to CYPs expression and regulation by interacting with NR biological functions [54]. The 2, 3, 7, 8-tetrachlorodibenzo-*p*-dioxin regulates CYP1A1 via an ubiquitin-dependent 26S proteasomal degradation-mediated downregulation of the AhR [219]. In addition, PXR is a target of the ubiquitin-signaling pathway, and phosphorylation controls PXR biologic function [220,221,222]. Much more interesting, however, is the possible critical role of the 26S proteasome as a modulator of CAR functional activity [223]. The ubiquitin proteasome was proved to be involved in the regulation of cytosolic proteins indirectly affecting CAR responsiveness to PB and PB-like compounds [224]. All this strengthened our idea to measure the effects of PB on the cattle 26S proteasome complex. The significant increase we observed in our experimental conditions suggested that cattle proteasome activity is activated in the presence of PB; moreover, it participated in targeting gene activation (*CYP2B22* and *CYP3A28* at first) in response to CAR ligands and inducers such as PB, and likewise to humans [223]. Such a hypothesis is fascinating, but needs challenging molecular confirmatory studies (e.g., using 26S proteasome inhibitors) to confirm whether such a significant increase is either nonspecific and compensatory or a true biomolecular response to PB exposure, occurring through a 26S proteasome and CAR interaction, also in light of the controversial regulatory mechanisms hypothesized for bovine CYP3As and involving CAR and PXR [65,66]. Despite this, the present results represent one of the very few cases in which the proteasome activity was measured in domestic animals, and particularly in cattle; moreover, to the best of our knowledge, this was the first study in which the 26S proteasome activity was measured in bovines administered a prototypical DME inducer such as PB.

## 4. Materials and Methods

### 4.1. Chemicals and Antibodies

Bovine serum albumin, glucose 6-phosphate, glucose 6-phosphate dehydrogenase, NADP^+^, 7-hydroxycoumarin (umbelliferone), and 4-aminophenol were obtained from Boehringer Mannheim GmbH (Mannheim, Germany). Phenobarbital sodium salt and all other reagents used for the measurement of catalytic activities were obtained from Sigma-Aldrich (St. Louis, MO, USA). Testosterone (TST), androstenedione, 6β- and 16β-hydroxytestosterone came from Steraloids (Newport, RI, USA). HPLC-grade methanol, acetonitrile, and dichloromethane were purchased from J.T. Baker (Phillipsburg, NJ, USA). Water was obtained from a Milli-Q ultrapurification system (Millipore Corporation, Milan, Italy). Antibodies raised against human CYP1A1/1A2 and CYP3A4 were obtained from Oxford Biomedical Research (Oxford, MI, USA); antirat CYP2B1 from Daiichi Pure Chemicals (Chuo-ku, Tokyo, Japan); antihuman CYP2C8/9/19 and antirat CYP2E1 from Chemicon International (Temecula, CA, USA); antihuman FMO1 and 3 were from Santa Cruz Biotechnology (Heidelberg, Germany); and anti β-actin (ACTB) and anti-calnexin (loading controls) were from Abcam (Cambridge, UK) and Santa Cruz Biotechnology (Heidelberg, Germany), respectively. All the antibodies were polyclonal, and preliminary studies confirmed their cross-reactivity with bovine microsomal proteins. Nitrocellulose membrane hybond-ECL and the ECL Western blotting analysis system were obtained from GE Healthcare (Piscataway, NJ, USA). Horseradish peroxidase-conjugated secondary antibodies were from Bio-Rad (Segrate, Milan, Italy). Chloroform, isopropyl alcohol, and ethyl alcohol were obtained from Thermo Electron Corporation (Waltham, MA, USA), whereas the TRIzol^TM^ reagent and agarose were from Life Technologies (Monza, Milan, Italy). All the other reagents were of molecular biology grade. The High-Capacity cDNA Reverse Transcription Kit and Power SYBR Green PCR Master Mix were obtained from Applied Biosystems (Foster City, CA, USA). Oligonucleotide primers were synthesized by Life Technologies (Monza, Milan, Italy).

### 4.2. Animals and Treatment

Seven male 10-month-old Friesian cattle were obtained from local farms. After a 1-month acclimation period, the animals were weighed (average weight 304 ± 16 kg) and randomly divided into 2 experimental groups; i.e., untreated control (UT, *n* = 3), and PB-treated (PB, *n* = 4) cattle; these latter animals were given PB (sodium salt, dissolved in water) by gavage at a dose of 18 mg/kg bw/day for 7 days. The animals were slaughtered 24 h after the last treatment. At the slaughterhouse, after exsanguination, the liver lobe was removed, and small aliquots for total RNA extraction (about 200 mg each) were collected in sterility, immediately snap-frozen in liquid nitrogen, and a posteriori stored at −80 °C until analysis. The remaining part of the lobe was cut into specimens, washed in chilled isotonic 1.15% KCl, wrapped in aluminium foil, put on ice, and brought to the laboratory where they were processed within two hours of tissue collection.

### 4.3. Preparation of Subcellular Fractions

Liver microsomal and cytosolic subcellular fractions from each animal were isolated by differential centrifugation, rapidly frozen in liquid nitrogen, and stored at −80 °C until use, as detailed elsewhere [10]. The protein content was determined with the Lowry’s method [225], using bovine serum albumin as the standard.

### 4.4. Enzyme Assays

#### 4.4.1. Spectral Measurements and Microsomal NAD(P)H Electron-Transferring Reductase Activities

The cytochromes P450 content was assayed as the carbon monoxide difference spectrum (450–490 nm) of sodium dithionite-reduced microsomal suspensions with an extinction coefficient of 90 mM cm^−1^ [226]. The content of cytochrome *b*_5_ was determined by the method of Lake (1987) as the difference spectrum (425–409 nm) of NADH-reduced vs. nonreduced microsomes, using an extinction coefficient of 185 mM cm^−1^ [227]. The extent of metyrapone binding was determined on microsomal suspensions as the difference between the absorption maximum at around 446 nm and the value at 490 nm (extinction coefficient 52 mM cm^−1^), based on the method of Liu and Franklin (1985) [228] with modifications described in detail in a previous paper [10]. The activities of NADPH CYP reductase and NADH cytochrome *b*_5_ reductase were measured by monitoring the cofactor-mediated reduction of cytochrome c at 550 nm [76].

#### 4.4.2. Cytochrome P450 Monooxygenases

The *O*-dealkylations of 7-ethoxyresorufin (2 µM), 7-methoxyresorufin (5 µM), 7-pentoxyresorufin (5 µM), or 7-benzyloxyresorufin (5 µM) were assayed fluorometrically with NADPH 1 mM and 0.2–0.3 mg protein by measuring the rate of resorufin formation (Ex: 530 nm; Em: 590 nm) [97]. The *N*-(*O*)demethylation assays used a NADPH-generating system, 1 mg protein, and the following substrates: aminopyrine (5 mM), benzphetamine (1 mM), chlorpheniramine (1 mM), erythromycin (1 mM), ethylmorphine (6 mM), monensin (0.25 mM), or triacetyloleandomycin (TAO, 0.3 mM) [10,229,230]. After quenching the reaction, the amount of the released formaldehyde was quantitated by a fluorometric method (Ex: 530 nm; Em: 590 nm) [231]. The *O*-demethylation of 7-ethoxy-4-trifluoromethylcoumarin (7-EFMC, 75 µM), and the *O*-demethylation of 7-methoxy-4-trifluoromethylcoumarin (7-MFMC, 50 µM), were measured fluorometrically by continuously monitoring the formation of 7-hydroxy-4-trifluoromethylcoumarin [232] as described elsewhere [230]. The *O*-demethylation of 7-ethoxycoumarin (0.8 mM) was determined as reported by Dent et al. (1976) [233], while coumarin 7-hydroxylase activity was assayed according to Van Iersel et al. (1994), using 10 µM substrate, 0.2 mg protein and NADPH 1mM [234]; in either case, the formation of hydroxycoumarin was monitored fluorometrically (Ex: 380 nm; Em: 452 nm). The method of Nebert (1978) [235] was used to measure the rate of benzo[a]pyrene (0.2 mM) hydroxylation, with modifications described in detail elsewhere [236]. The rate of the in vitro 4-hydroxylation of aniline (5 mM) was determined by measuring the formation of 4-aminophenol according to Ugazio et al. (1991) [237]. The hydroxylation of 4-aminophenol (0.2 mM) was assayed based on the method of Reinke and Moyer (1985) [238] with modifications detailed in a previous paper [10]. Finally, the hydroxylation of TST was measured according to the HPLC method published by Purdon and Lehman-McKeeman (1997) [239], with minor modifications described in Pegolo et al. (2010) [240].

#### 4.4.3. Flavin-Containing Monooxygenases

The catalytic activity of FMOs was determined by measuring the substrate-mediated NADPH oxidation under the assay conditions described by Dixit and Roche (1984) [241] to minimize the CYP-mediated contribution to NADPH oxidation, using 0.2–0.4 mg microsomal protein and either methimazole (MTZ, 1 mM) or ethylenethiourea (ETU, 1 mM) as substrates; in both cases, the enzyme activity was measured in the presence of octylamine (1 mM), a known FMO activator [242].

#### 4.4.4. Hydrolytic Enzymes

Microsomal CES were assayed with different esters. The hydrolysis of *p*-nitrophenylacetate (0.33 mM, PNP) or indophenylacetate (0.33 mM, IPA) were determined following the procedures described by Nousiainen et al. (1984) [243] and Zemaitis and Greene (1979) [244], respectively. For the measurement of α-naphtylacetate (ANA) activity, the reaction mixture included potassium phosphate buffer (50 mM, pH 7.4) and protein (0.004–0.005 mg). After equilibration at 30 °C, the reaction was started with the substrate (0.66 mM dissolved in ethanol), and product formation (α-naphthol) was followed at 331 nm for 3 min. The procedure of Hasegawa and Hammock (1982) [245] was followed for cytosolic EH measurement, using *trans*-stilbene oxide (TSO, 50 µM) as the substrate and 0.25 mg protein. The rate of substrate disappearance at 229 nm was taken as an index of EH activity.

#### 4.4.5. Conjugative Enzymes

UDP-glucuronosyltransferase activity was determined according to Antoine et al. (1988) [246] on 0.25% Triton-X100 activated microsomes (0.13 mg/mL) using 0.2–0.4 mg protein and different substrates; i.e., 1-naphthol (0.3 mM), *p*-nitrophenol (0.3 mM), chloramphenicol (0.25 mM), or dexamethasone (2.5 mM). The activities of cytosolic glutathione (GSH) *S*-transferase (GST) families (α, µ, π) toward 1-chloro-2,4-dinitrobenzene (CDNB, overall substrate), 3,4-dichloronitrobenzene (DCNB, µ), or ethacrynic acid (ETA, π) were assayed by measuring the formation of the respective GSH adducts under assay conditions detailed elsewhere [8]. Cumene hydroperoxide (CUH) was used for the GSTα assay according to Di Simplicio et al. (1989) [247].

#### 4.4.6. Proteasome Activity

Proteasome chymotrypsin-like activity in liver extracts was assayed by in continuo monitoring of the production of 7-amino-4-methylcoumarin (AMC) from the fluorogenic peptide Suc-LLVY-amc according to the procedure by Cascio et al. (2002) [248].

### 4.5. Sodium Dodecyl Sulfate (SDS)-Polyacrylamide Gel Electrophoresis and Immunoblotting

Microsomal proteins (range 10–100 μg) were separated by 10% SDS-polyacrylamide gel electrophoresis in a Bio-Rad Miniprotean cell (Hercules, CA, USA) and transferred to hybond-ECL nitrocellulose membranes according to Nebbia et al. (2003) [10]. Membranes were then firstly probed with appropriate dilutions, previously identified in a setup procedure, of goat antihuman CYP1A1/1A2, rat antihuman CYP2B6, rabbit antihuman CYP2C8/9/19, rabbit antihuman CYP2E1, rabbit antihuman CYP3A4, mouse antihuman FMO1, goat antihuman FMO3, rabbit antibovine ACTB, and rabbit antihuman calnexin antibodies. Therefore, they were incubated with suitable peroxidase-conjugated secondary antibodies. Proteins were detected using ECL Western blotting detection reagents. Immunoblot bands were visualized by using the ChemiDocMP System (Bio-Rad, Segrate, Milan, Italy). Integrated optical densities of immunopositive bands were calculated by means of the Bio-Rad software Quantity One (version 4.5.2; Bio-Rad, Segrate, Milan, Italy). The relative density of each individual protein band was normalized to that of the corresponding loading control (ACTB).

### 4.6. Total RNA Isolation, Reverse Transcription, and Quantitative Real Time RT-PCR (qPCR)

Total RNA was isolated from frozen liver aliquots using the TRIzol^TM^ reagent and according to the manufacturer’s instructions, as previously reported by Giantin et al. (2008) [189]. Total RNA concentration and quality were checked using the Nanodrop ND-1000 spectrophotometer (Labtech France, Paris, France). The RNA quality was estimated using the 260/280 and 260/230 nm absorbance ratios and confirmed by denaturing agarose gel electrophoresis.

A reverse transcription was performed by using the High-Capacity cDNA Reverse Transcription Kit and 2 μg of total RNA (for a final reaction mixture volume of 20 μL), following the manufacturer’s procedure. The reaction was performed in a 96-well GeneAmp PCR System 9700 (Applied Biosystems) in the following conditions: 10 min at 25 °C and 2 h at 37 °C. Complementary DNA was then stored at −20 °C until use.

Bovine mRNA sequences of target genes were obtained from the GenBank and Ensembl Genome Browser web sites (http://ncbi.nlm.nih.gov and http://www.ensembl.org respectively, accessed on 21 May 2020). Primers sequences for qPCR (Table S4) were designed using Primer Express Software (version 2.0; Applied Biosystems). Primers concentrations were optimized in the 300 to 900 nM range. Melting curve analysis and agarose gel electrophoresis confirmed the amplification of a single amplicon of the expected size, as well as the absence of primer dimers and genomic DNA amplification. Calibration curves, using a 10-fold serial dilution of a cDNAs pool, revealed PCR efficiencies close to 100%; therefore, the ΔΔCt method [249] was used to analyze data, expressed as the fold change compared with UT. The β-actin was considered as the internal (reference) control gene. The qPCR reaction was performed on 5 μL, out of a 25 μL final volume, of 20-fold-diluted cDNA by using an ABI Prism 7000 Sequence Detection System (Applied Biosystems), using standard PCR conditions.

### 4.7. Statistical and Data Analysis

Data were expressed as mean values ± S.D. The statistical analysis (GraphPad Software Inc., San Diego, CA, USA; version 8.0.2) was performed by using an unpaired *t*-test, with a *p*-value of at least <0.05 considered as statistically significant.

## 5. Conclusions

Overall, this work provided the first and almost complete characterization of PB-dependent changes in DME catalytic activities in cattle liver, measured by using the most commonly used probe substrates. Confirmatory qPCR and immunoblotting investigations were also carried out for some CYPs and FMOs. The barbiturate increased CYP content and the extent of metyrapone binding, as expected. A consistent and univocal response; i.e., an upregulation of both mRNA and protein levels, as well as of the related enzyme activities, was observed for known PB-targeted CYPs; i.e., CYP2B, 2C, and 3A, but also and surprisingly for CYP2E1. Less clear-cut and sometimes contradictory results, when compared to the overall comparative knowledge about PB’s inducing properties, were obtained for CYP1A. For the first time, we also measured the effect of PB on the foremost FMOs (1 and 3), and a decrease in the in vitro metabolism of probe substrates was observed.

On the contrary, PB had no effect on the considered hydrolytic and phase II DMEs. Nevertheless, caution should be exercised when comparing and interpreting the present data with those referring to humans and rodents; as a matter of fact, we still do not have fully reliable species-specific substrates for each of the abovementioned DMEs; moreover, the knowledge of the biomolecular mechanisms involved in phase II DME expression, regulation, and biological activity is much lower than that of CYPs, which is still limited from a comparative point of view. Therefore, we discourage the direct extrapolation of data from human and rodents to bovines. Finally, for the first time, we measured the 26S proteasome activity in PB-treated cattle, and the increase we observed could be indicative of a role of this post-translational event in the regulation of cattle DMEs, especially (but not exclusively) of CYPs.

Overall, the obtained results increased the knowledge of hepatic drug metabolism in this important food-producing species. They confirmed that differences in DME expression and activity existed between bovines, humans, and rodents (and other veterinary species as well), thus confirming once more the need for an extensive characterization and understanding of comparative molecular mechanisms involved in expression, regulation, and function of DMEs. Nowadays, such a concept is of extreme importance, especially to avoid extrapolation of data referring to kinetics, efficacy, and safety of xenobiotics from one species to another, with increasing risks for the animal itself and for consumers.

## Data Availability

Not applicable.

## Supplementary Materials

The following supporting information can be downloaded at: https://www.mdpi.com/article/10.3390/ijms23073564/s1.
