# Supplementary material for: Induction by Phenobarbital of Phase I and II Xenobiotic-Metabolizing Enzymes in Bovine Liver: An Overall Catalytic and Immunochemical Characterization
Source: Int J Mol Sci. 2022 Mar 24;23(7):3564. doi: 10.3390/ijms23073564 (PMC8998613; doi:10.3390/ijms23073564)
Supplement: Supplementary file 1 [file ijms-23-03564-s001.zip › ijms-1618089-supplementary.pdf]

International Journal of *Molecular Sciences*

MDPI

Supplementary materials

Induction by Phenobarbital of Phase I and II Xenobiotic-Metabolizing Enzymes in Bovine Liver: An Overall Catalytic and Immunochemical Characterization

Michela Cantiello [1,†,‡], Monica Carletti [1,†,§], Mery Giantin [2], Giulia Gardini [1], Francesca Capolongo [2], Paolo Cascio [1], Marianna Pauletto [2], Flavia Girolami [1], Mauro Dacasto [2,*] and Carlo Nebbia [1,*]

[1] Department of Veterinary Sciences, University of Turin, 10095 Grugliasco, Italy; michela.cantiello@gmail.com (M.C.); monica.carletti@irta-ricerche.it (M.C.); giulia.gardini@unito.it (G.G.); paolo.cascio@unito.it (P.C.); flavia.girolami@unito.it (F.G.)

[2] Department of Comparative Biomedicine and Food Science, University of Padua, 35020 Agripolis Legnaro, Italy; mery.giantin@unipd.it (M.G.); francesca.capolongo@unipd.it (F.C.); marianna.pauletto@unipd.it (M.P.)

* Correspondence: mauro.dacasto@unipd.it (M.D.); carlo.nebbia@unito.it (C.N.)

† These authors contributed equally to this work.

‡ Current address: Eurofins Biopharma Services, 33000 Bordeaux, France.

§ Current address: IRTA Ricerche s.r.l., 10093 Collegno, Italy.

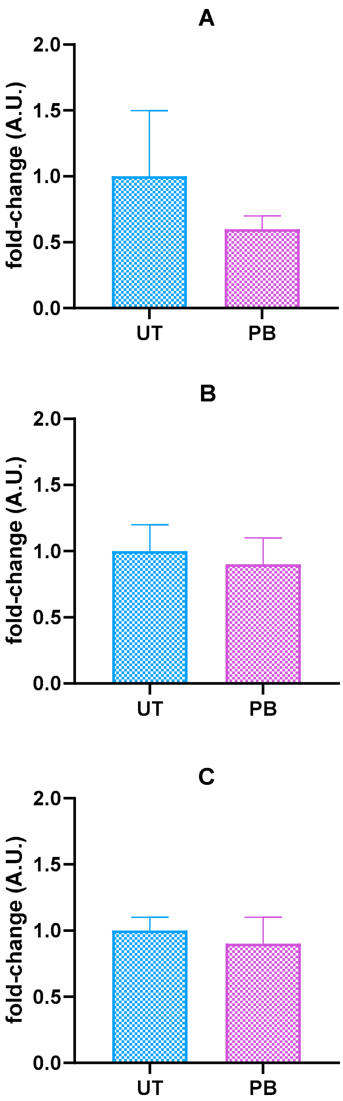

Figure S1. Hepatic nuclear receptors mRNA levels in untreated control (UT, n=3) and phenobarbital-treated cattle (PB, n=4). Total RNA was extracted from cattle liver aliquots; then, constitutive androstane receptor (*CAR*) (A), pregnane X receptor (*PXR*) (B), and retinoid X-receptor alpha (*RXRα*) (C) mRNA levels were measured by using a quantitative real-time RT-PCR (qPCR) approach. Data (arithmetic means ± SD) are expressed as n-fold change (arbitrary units, A.U.), normalized to ΔΔCt mean value of β-actin (ACTB, the chosen internal control gene, ICG), to whom an arbitrary value of 1 was assigned.

Table S1. Cytochromes P450 and b_5 content, NADPH cytochrome *c* (P450) reductase and NADH cytochrome b_5 reductase activities, and extent of metyrapone binding in untreated control (UT, n = 3) and phenobarbital-treated (PB, n = 4) cattle.

Parameter	UT	PB
total CYP†	0.80 ± 0.10	1.63 ± 0.24**
total cytochrome b_5†	0.35 ± 0.13	0.45 ± 0.08
NADPH cytochrome *c* (P450) reductase‡	81.67 ± 10.10	87.38 ± 13.29
NADH cytochrome b_5 reductase‡	1077.00 ± 108.67	383.50 ± 75.79***
metyrapone binding†	0.12 ± 0.03	0.96 ± 0.23**

Data are expressed as means ± SD. CYP: cytochrome P450. ** $p < 0.01$; *** $p < 0.001$ (unpaired *t*-test). † nmoles/mg protein. ‡ nmoles/min·mg $protein^{-1}$.

Table S2. Cytochrome P450 (CYP) 1A, CYP2A, and CYP2E1 protein expression and *in vitro* metabolism of marker substrates in untreated control (UT, n = 3) and PB-treated (PB, n = 4) cattle.

Parameter	UT	PB
CYP1A expression†	214.00 ± 33.50	139.50 ± 25.60*
7-ethoxyresorufin deethylation‡	209.72 ± 40.71	138.15 ± 33.01*
7-methoxyresorufin deethylation‡	43.60 ± 1.18	165.57 ± 36.59**
benzo[*a*]pyrene hydroxylation§	1.22 ± 0.12	2.19 ± 0.35**
7-ethoxycoumarin deethylation#	0.71 ± 0.15	1.41 ± 0.29*
CYP2A expression†	N.A.	N.A.
coumarin hydroxylation‡	104.00 ± 41.00	95.00 ± 75.00
CYP2E1 expression†	125.50 ± 34.40	363.30 ± 192.80
Aniline hydroxylation#	0.65 ± 0.10	1.37 ± 0.33*
4-Aminophenol hydroxylation#	0.28 ± 0.05	0.79 ± 0.14**

Data are expressed as means ± SD. * $p < 0.05$; ** $p < 0.01$ (unpaired *t*-test). † arbitrary units (A.U.). ‡ pmoles/min·mg $protein^{-1}$. § µg quinine/mL. # nmoles/min·mg $protein^{-1}$. N.A. not available.

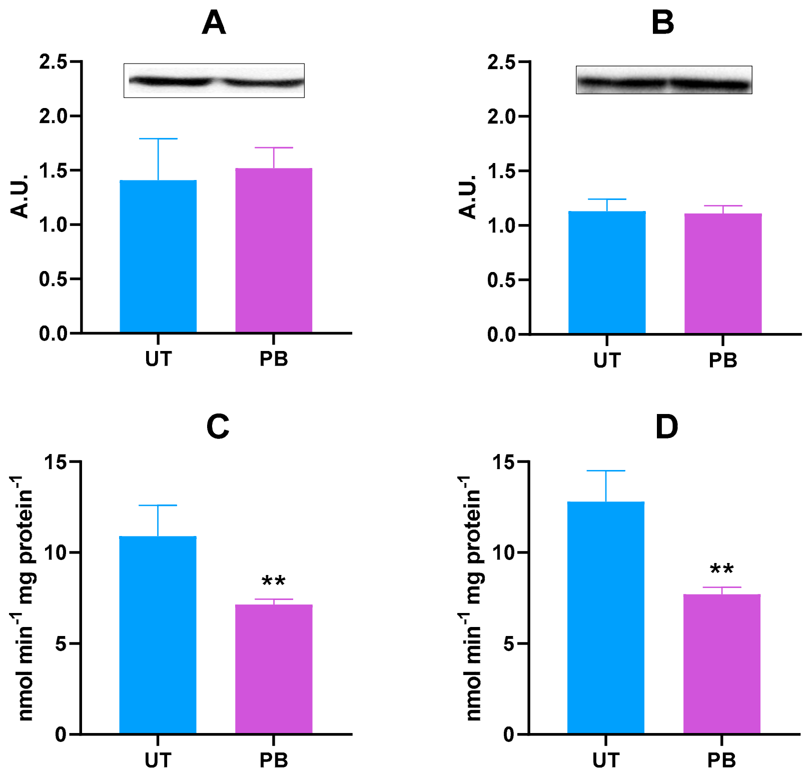

Figure S2. Hepatic flavin monooxygenase 1 and 3 (FMO1 and FMO3) protein expression (A-B) and *in vitro* metabolism of marker substrates ethylenethiourea and methimazole (C-D) in untreated control (UT, n=3) and phenobarbital-treated (PB, n=4) cattle. A.U., arbitrary units; ETU, ethilenethiourea; MTZ, methimazole. Data are expressed as arithmetic means ± SD. ** $p < 0.01$; *** $p < 0.001$ (unpaired *t*-test).

Table S3. *In vitro* metabolism of carboxylesterase and epoxide hydrolase marker substrates in untreated control (UT, n = 3) and phenobarbital-treated (PB, n = 4) cattle.

Parameter	UT	PB
APA hydrolysis∞	5.55 ± 0.78	8.18 ± 1.08*
IPA hydrolysis#	334.87 ± 83.54	368.72 ± 18.56
PNP hydrolysis∞	5.65 ± 2.39	6.69 ± 0.90
trans-stilbene oxide epoxide hydrolysis#	2.46 ± 0.08	1.95 ± 0.36

APA: α-naphtylacetate; IPA: indophenyl acetate; PNP: *p*-nitrophenylacetate. Data are expressed as means ± SD. * $p < 0.05$ (unpaired *t*-test). ∞ µmoles/min·mg protein^{-1}. # nmoles/min·mg protein^{-1}.

Table S4. Gene acronyms, GenBank accession numbers, qPCR primers (references and oligonucleotide sequences) and amplicon size.

Gene	GeneBank ID	Primer reference	5′ → 3′ primer sequence	Amplicon size (bp)
ACTB	NM_173979	Giantin et al., 2008	F: GTCGACACCGCAACCAGTT<br>R: AAGCCGGCCTTGCACAT	85
CYP2B22	NM_001075173	Giantin et al., 2008	F: GCGGACCTCATCCCCATT<br>R: GTGCCCTTGGGAAGGATGT	80
CYP2C18	NM_001076051	Giantin et al., 2008	F: ATGTTAAGAACATTGGCAAATCCTT<br>R: GGCCATAGGTGTTTGAGAGATTG	51
CYP2C31	XM_600421	Giantin et al., 2008	F: TCCCAAGGGCACAACCATA<br>R: CCTTGCCATCGTGCAGG	56
CYP2C42	XM_612374	Giantin et al., 2008	F: TCCCTGGACATGAACAACCC<br>R: TTGTGCTTTTCCTGTTCCATCTT	71
CYP3A	NM_174531	Cantiello et al., 2009	F: GCCAGAGCCCGAGGAGTT<br>R: GCAGGTAGACGTAAGGATTTATGCT	77
PXR	NM_001103226	Cantiello et al. 2009	F: TGAAGGCCTACATCGAGTTCAAC<br>R: GGCCATGATCTTCAGGAACAA	68
CAR	NM_001079768	Cantiello et al. 2009	F: GAAGGACATGATCCTATCGACAGA<br>R: CGTCGCTGGGCCTGTCT	63
RXRα	XM_881943	Cantiello et al. 2009	F: GCCTCAATGGTGTCCTCAAAG<br>R: AGCTGTACACCCCGTAGTGCTT	120

ACTB: β-actin; bp: base pairs; *CAR*, constitutive androstane receptor; *CYP2B22*, cytochrome P450 2B22; *CYP2C18*, cytochrome P450 2C18; *CYP2C31*, cytochrome P450 2C31; *CYP2C42*, cytochrome P450 2C42; *CYP3A*, cytochrome P450 3A; *PXR*, pregnane X receptor; qPCR, quantitative Real time RT-PCR; *RXRα*, retinoid X-receptor alpha.
